# Lifelong cytomegalovirus and early‐LIFE irradiation synergistically potentiate age‐related defects in response to vaccination and infection

**DOI:** 10.1111/acel.13648

**Published:** 2022-06-03

**Authors:** Jason L. Pugh, Christopher P. Coplen, Alona S. Sukhina, Jennifer L. Uhrlaub, Jose Padilla‐Torres, Tomonori Hayashi, Janko Nikolich‐Žugich

**Affiliations:** ^1^ Department of Immunobiology University of Arizona College of Medicine Tucson Arizona USA; ^2^ Arizona Center on Aging University of Arizona College of Medicine Tucson Arizona USA; ^3^ Graduate Interdisciplinary Program in Genetics University of Arizona Tucson Arizona USA; ^4^ Radiation Effects Research Foundation Minami‐Ku Hiroshima Japan; ^5^ BIO5 Institute University of Arizona Tucson Arizona USA

**Keywords:** aging, cytomegalovirus, DNA damage, T cells, vaccination, West Nile virus

## Abstract

While whole‐body irradiation (WBI) can induce some hallmarks of immune aging, (re)activation of persistent microbial infection also occurs following WBI and may contribute to immune effects of WBI over the lifespan. To test this hypothesis in a model relevant to human immune aging, we examined separate and joint effects of lifelong latent murine cytomegalovirus (MCMV) and of early‐life WBI over the course of the lifespan. In late life, we then measured the response to a West Nile virus (WNV) live attenuated vaccine, and lethal WNV challenge subsequent to vaccination. We recently published that a single dose of non‐lethal WBI in youth, on its own, was not sufficient to accelerate aging of the murine immune system, despite widespread DNA damage and repopulation stress in hematopoietic cells. However, 4Gy sub‐lethal WBI caused manifest reactivation of MCMV. Following vaccination and challenge with WNV in the old age, MCMV‐infected animals experiencing 4Gy, but not lower, dose of sub‐lethal WBI in youth had reduced survival. By contrast, old irradiated mice lacking MCMV and MCMV‐infected, but not irradiated, mice were both protected to the same high level as the old non‐irradiated, uninfected controls. Analysis of the quality and quantity of anti‐WNV immunity showed that higher mortality in MCMV‐positive WBI mice correlated with increased levels of MCMV‐specific immune activation during WNV challenge. Moreover, we demonstrate that infection, including that by WNV, led to MCMV reactivation. Our data suggest that MCMV reactivation may be an important determinant of increased late‐life mortality following early‐life irradiation and late‐life acute infection.

## Graphical abstract

Early‐life sublethal radiation increases cytomegalovirus (CMV) reactivation and depresses CMV immunity, making older mice vulnerable to additiona bouts of CMV reactivation and death upon third‐party infection.

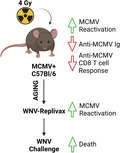

**Abreviations**
Gy: gray (unit of radiation);
MCMV: murine cytomegalovirus;
RWN: Replivax West Nile;
WBI: whole body irradiation;
WNV: West Nile Virus

## 1 INTRODUCTION

Susceptibility to infectious disease increases with age, making it one of the leading causes of death in people over 65 (CDC, 2010; NCHS, 2013). Aging is associated with a variety of immune defects affecting both innate and adaptive parts of immunity (Rev. in Nikolich‐Zugich, 2018). Adaptive immunity age‐associated defects include decreased naïve T‐cell numbers (Rudd et al., 2011; Smithey et al., 2012, proliferation (Haynes et al., 1999; Jiang et al., 2007; Jiang et al., 2013; Smithey et al., 2011) and function (Deng et al., 2004; Haynes et al., 1999; Jiang et al., 2007; Jiang et al., 2013; Smithey et al., 2011) along with reduced pathogen‐specific antibody production, somatic hypermutation and protective efficacy (Frasca et al., 2004; Frasca et al., 2008). Theories of biological aging suggest that somatic cells, including immune cells, accumulate age‐related defects, leading to impaired maintenance, function and, where applicable, self‐renewal; DNA damage‐related senescence is one of the prominent factors implicated in these defects (Campisi, 2005; d'Adda di Fagagna, 2008; Hasty et al., 2003; Jeyapalan et al., 2007; LLopez‐Otin et al., 2013), but our understanding of long‐term effects of DNA damage upon immune function in the old age in vivo is limited.

In addition, immune aging was hypothesized to be accelerated by the presence of life‐long latent infections that impose a potential life‐long burden on the immune system. One of the most prevalent latent viruses, the cytomegalovirus (CMV), has been associated in some (Cicin‐Sain et al., 2012; Mekker et al., 2012; Smithey et al., 2018), but not other (Marandu et al., 2015), studies to altered, suboptimal immune responses and increased all‐cause mortality in both animal models and humans (Cicin‐Sain et al., 2012; den Elzen et al., 2011; Kilgour et al., 2013; Simanek et al., 2011; Staras et al., 2006; Trzonkowski et al., 2003; Wang et al., 2010), although the mechanistic effects of CMV upon the aging immune system remain only partially understood. The most remarkable effect of CMV upon the immune system manifests in “memory inflation”—the presence of highly differentiated CMV‐specific effector memory‐phenotype T cells that increase in number throughout life (Karrer et al., 2003; Welten et al., 2013).

Whole‐body gamma irradiation (WBI) results in systemic DNA damage (Simpkin, 1999) and hematopoietic lineage cell death in a dose‐dependent manner (Anderson, 1976; Nias, 1990). At WBI doses in the 0.5–4 Gray (Gy) range, hematopoietic cell populations are dramatically depleted. Barring severe host infection and death, in young organisms surviving immune cells divide rapidly and eventually repopulate to pre‐irradiation levels (Anderson, 1976). While the kinetics of immune repopulation has been described, long‐term immune system performance following repopulation remains relatively uncharacterized. Hiroshima and Nagasaki bomb survivors display certain hallmarks of increased or premature immune aging (Hayashi et al., 2005; Kusunoki & Hayashi, 2008; Nakachi et al., 2004; Yamaoka et al., 2004). However, little is known about the influence of life‐long CMV on the immunity of radiation survivors. In our previous study (Pugh et al., 2016), we have shown that a single exposure of C57BL/6 (B6) mice to ionizing radiation of up to 4Gy in youth (equivalent to about 2Gy in humans) does not leave permanent scars on the immune system during aging, and that the animals responded well to vaccination and resisted subsequent challenge as well as their non‐irradiated counterparts.

However, experiments in specific pathogen‐free animals can often be misleading, and it is important to consider the impact of microbial exposure in mimicking human physiology. Human cytomegalovirus (CMV) is one of the most prevalent persistent viruses. Often acquired in early life (Krstanovic et al., 2021), CMV results in a life‐long latent infection with opportunistic lifelong reactivation (Balthesen et al., 1994; Taylor‐Wiedeman et al., 1991). CMV exhibits a profound and cumulative impact on host immunity (Nikolich‐Zugich et al., 2020), modulating an exceptionally large number of immune parameters (Brodin et al., 2015). To model the potential influence and interdependence of WBI, CMV, and aging on immunity, we here employed murine cytomegalovirus (MCMV) in a mouse model of WBI and natural aging. We tested vaccination and immunity in old age using the single‐cycle live vaccine RepliVAX West Nile (R‐WN) (Widman et al., 2009), followed by challenge with a potentially lethal dose of live West Nile virus (WNV), as in our previous work (Pugh et al., 2016). Though neurotropic in later‐stages of infection, WNV initially travels to and replicates in a variety of organs, evoking a systemic immune response (Brien et al., 2007; Brien et al., 2009).

Contrary to the results with uninfected irradiated mice, where vaccine and WNV‐specific immunity, including T cell and antibody responses, were not substantially affected by WBI dose in youth, we found that in young adult animals carrying CMV, WBI induced immediate and clear reactivation of CMV. Mice carrying CMV and exposed to WBI in youth also exhibited signs of reduced immunity against CMV in the old age, as measured by reduced Th1 cytokine expression levels and percentages of cytokine‐producing cells, increased expression of PD‐1 on CMV‐specific cells and reduced total anti‐CMV antibody titers. In late life, CMV‐positive animals irradiated in youth exhibited higher mortality following WNV challenge despite being vaccinated by R‐WN, whereas animals exposed to WBI only or CMV only were fully protected, just as the control, unirradiated and uninfected animals. This reduced survival was associated with opportunistic MCMV reactivation during WNV challenge, likely resulting in a reduced ability of the irradiated immune system to deal with both the reactivated CMV and the WNV primary infection.

## 2 RESULTS

### 2.1 Hypotheses and experimental design

If DNA‐damage related senescence is a causative factors in immune aging, we hypothesized that WBI would increase aging immune phenotypes in a dose‐dependent manner. Further, if the effects of DNA damage are potentiated by cellular turnover in a causative manner, the combination of MCMV and WBI would be expected to result in an additive immune aging effect.

**FIGURE 1:**
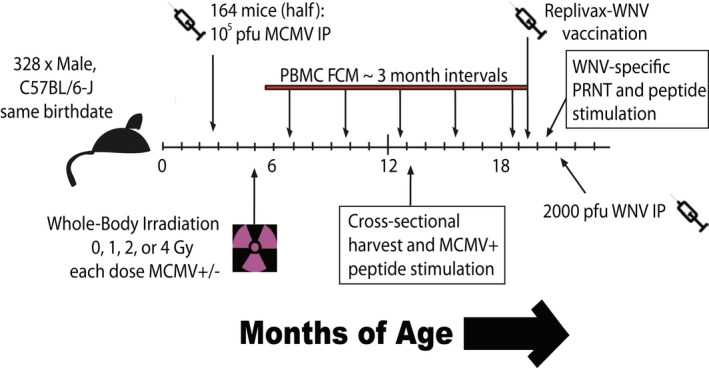
Experimental design of longitudinal cohorts. Age‐matched, adult, male, C57BL/6 mice were divided into those receiving MCMV(+), and those remaining uninfected with MCMV(−), for life. Following a 60‐day rest, mice in each group were then further divided into those receiving 0, 1, 2, or 4 gray (Gy) of WBI in a single dose. A cross‐sectional harvest of representative mice from each group was collected at 13 months of age, long after complete repopulation of immune cells. In addition, immune populations in peripheral blood were tracked at 3‐month intervals from WBI until 19 months of age by flow cytometry (FCM). At 19 months of age, mice were injected with 10^5^ pfu RWN vaccine IP. Immune function and antibody generation were assayed 45‐days post‐vaccination. 60 days post‐vaccination, mice were challenged with 2000 pfu WNV IP

In order to test these hypotheses, we employed the experimental strategy outlined in Figure 1. Age‐matched, adult, male, C57BL/6 mice were divided into mock‐infected and MCMV‐infected groups at 2.3 months of age. Following a 60‐day rest to allow for MCMV to establish stable latency, mice in each group were exposed to 0, 1, 2, or 4 Gray (Gy) WBI in a single dose. As would be expected from the WBI LD50/30 of this strain (37), no mice died from the exposure. 72 h post‐WBI, as well as at 13 and 19 months of age, 6 mice/group were analyzed cross‐sectionally for lymphoid cell depletion/death and repopulation in a WBI dose‐dependent manner. In addition, immune populations in peripheral blood were tracked at 3‐month intervals from WBI until 19 months of age. At 19 months of age, mice were injected with RWN vaccine, which we have previously shown to be protective from WNV in old mice (Uhrlaub et al., 2011). Following development of primary and memory immune responses (60 days post‐infection), we challenged mice with a potentially lethal dose of live WNV and determined survival. Following both vaccination (Day 7) and WNV challenge (Day 67), we also measured WNV‐specific adaptive immunity as described below. This design was replicated on two independent cohorts of mice separated by approximately 1 year, with comparable results.

### 2.2 High‐dose WBI in youth results in reduced survival from WNV challenge in old age only in mice with life‐long MCMV

**FIGURE 2:**
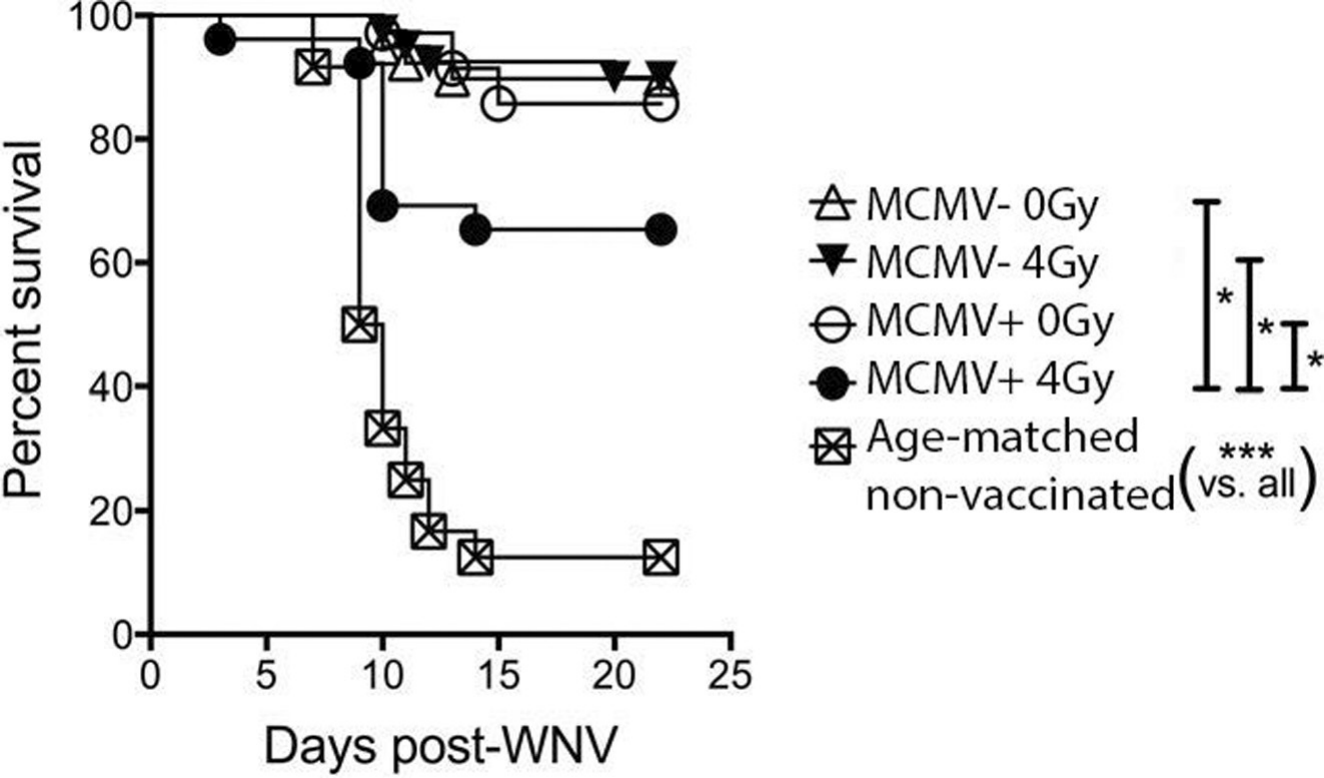
Additive effect of MCMV infection and WBI in youth on recall WNV survival in aged mice. Survival shown following RWN vaccination at approximately 19 months of age, and WNV challenge at approximately 21 months of age. Mice were infected with 2000 pfu WNV IP. Graph is a combination of both cohorts (no group statistically different between cohorts). Shown are results of individual comparisons from Kaplan–Meier tests. *N* ≥ 12 per group. Log‐rank test for trend = ****

Following vaccination and WNV challenge, the vast majority of mice in the MCMV(+) 0 Gy, MCMV(−) 4 Gy, and MCMV (−) 0 Gy (control) groups all survived WNV challenge. However, survival in MCMV(+) 4 Gy mice was significantly worse compared with either of the above three groups (Figure 2). Lower irradiation doses (1 & 2 Gy) had no significant impact on survival regardless of the presence of MCMV (Figure S1). Therefore, neither WBI alone nor CMV alone, administered in youth, could adversely affect the ability of an old organism to survive WNV challenge following vaccination.

### 2.3 High‐dose sub‐lethal WBI in youth does not significantly alter vaccination efficacy with age

**FIGURE 3:**
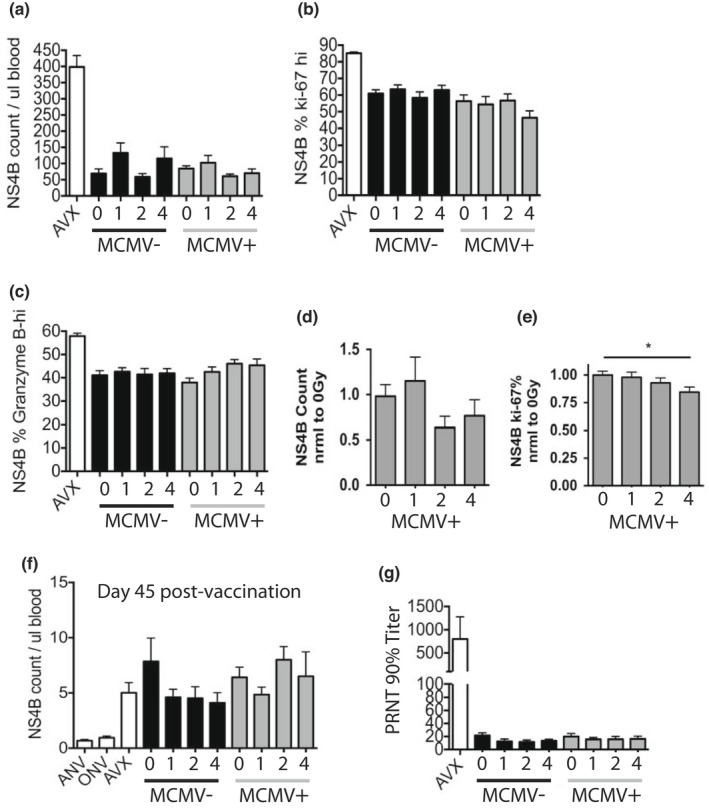
Vaccine response is equivalent in old mice exposed to WBI in youth. All mice injected with 10^5^ pfu RWN IP on the same day. AVX = adult vaccinated controls (5 months). ANV = adult non‐vaccinated controls, ONV = age‐matched old non‐vaccinated controls. All old groups ≥19 months. AVX, ANV, and ONV groups were MCMV(−), and not subjected to WBI. Numbers on x‐axis indicate dose of WBI in youth in Gy. All data from PBMC. (A‐D, H, I) shown from a single cohort, *n* ≥ 8 per group. (a) Counts of NS4B tet + CD8 T cells on day 7 post‐RWN. 1‐way Kruskal–Wallis without AVX control = NS. No significant Dunn's post‐test for MCMV, WBI extremes. (b) Percent of Ki‐67 hi, NS4B tet + CD8 T cells on day 7 post‐RWN. 1‐way Kruskal–Wallis without AVX control = NS. No significant Dunn's post‐test for MCMV, WBI extremes. (c) Percent of NS4B tet + CD8 T cells that are granzyme B‐hi on day 7 post‐RWN. 1‐way Kruskal–Wallis without AVX control = NS. No significant Dunn's post‐test for MCMV, WBI extremes. (d‐e) Data shown from combined cohorts normalized to 0Gy (no WBI) MCMV(+) mice in each group, *n* ≥ 15 per group. (d) Counts of NS4B+ CD8 T cells in MCMV+ mice on day 7 post‐RWN. 1‐way ANOVA = NS. Dunnet's multiple comparison not significant between M0 and M4. (e) Percent of Ki‐67‐hi in MCMV(+) mice on day 7 post‐RWN. 1‐way ANOVA = NS. Results of Dunnet's multiple comparison shown. (f) Counts of established memory NS4B tet + CD8 T cells on day 45 post‐RWN. (g) Maximum dilution of serum collected 45 days post‐RWN vaccination, that achieves 90% reduction of WNV plaques at 100 pfu. 1‐way Kruskal–Wallis without AVX control = NS. No significant Dunn's post‐test for MCMV, WBI extremes

To investigate how WBI and CMV could damage the immune system, we followed immune cell populations throughout life (gated as in Figure S2). Most populations were not significantly different across WBI doses by 19 months of age, at the time of vaccination, including B, NK‐T, and γδ (Figure S3). While NK cell counts at 19 months appeared the most altered by WBI in youth, NK cells are dispensable for survival from WNV (Shrestha et al., 2006). NS4b is the dominant CD8 T‐cell epitope responding to WNV and the RWN vaccine (Brien et al., 2007). At 7 days post‐vaccination, NS4B tetramer positive (NS4b+) CD8 T cells were equivalently abundant in groups receiving different WBI doses and also between MCMV(−) and MCMV(+) groups (Figure 3a, (Pugh et al., 2016)). Anti‐Ki‐67 antibody marks cells currently in the midst of any phase of cell cycle (G1,S,G2,M), but not those in interphase (G0) (Lopez et al., 1991). Ki‐67+ NS4b + cells were also found in similar frequencies across all groups at the peak of the vaccination response (Figure 3b, (Pugh et al., 2016)). The exception was the MCMV(+) 4Gy mice, which exhibited lower representation of these cells compared with their 0Gy counterparts when data from cohorts were combined (Figure 3e). NS4B+ cells also contained a similar proportion of Granzyme B^hi^ cells across all groups (Figure 3c, (Pugh et al., 2016)). In the memory phase (45 days post‐vaccination and just prior to challenge), numbers of NS4B+ memory CD8+ T cells were comparable with adult vaccinated controls across all WBI and CMV groups (Figure 3f). Finally, the analysis of sera at 45 days post‐vaccination revealed similar levels of neutralizing of WNV‐specific antibodies (as judged by the plaque reduction neutralizing titer [PRNT] assay), revealed that neither WBI dose in youth, nor life‐long MCMV status significantly altered the neutralizing potential of serum antibody generated by RWN vaccination in old mice (Figure 3g, (Pugh et al., 2016)), albeit the neutralizing titers were significantly lower compared with adult vaccinated animals (Figure 3g, (Pugh et al., 2016)) confirming our published work (Uhrlaub et al., 2011).

**FIGURE 4:**
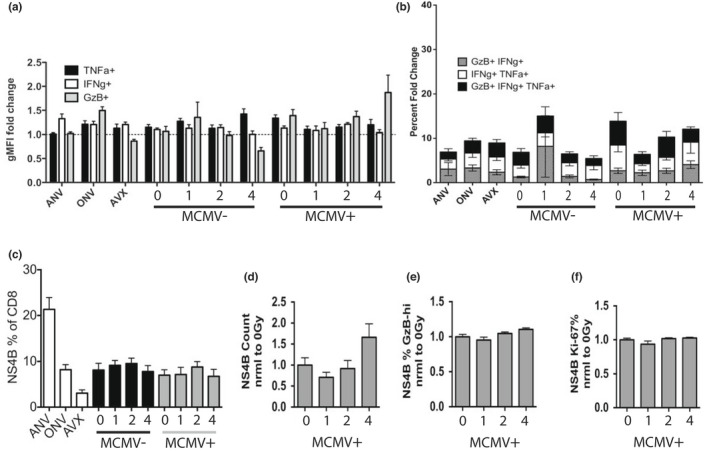
T‐cell function and responses to WNV challenge in mice exposed to WBI in youth. AVX = adult vaccinated controls, ANV = adult non‐vaccinated controls (5 months). ONV = non‐vaccinated mice, age‐matched to cohort (21 months). AVX, ANV, and ONV groups were MCMV(−), and not subjected to WBI. Numbers on x‐axis indicate dose of WBI in youth in Gy. M indicates mice that have had MCMV for life. (a‐b) Shown from a single cohort, *n* ≥ 10 per group, except ANV = 4. PBMC from mice on day 45 post‐RWN vaccination were isolated and stimulated with CD8‐specific WNV peptides for 5 h in the presence of brefeldin a. (a) Fold change (peptides / no peptides) of individual cytokine production by geometric MFI. 2‐way ANOVA: Between groups, *p*‐value = 0.0008, between cytokines = NS. 0Gy MCMV+ vs. 4Gy MCMV+ groups in Tukey multiple comparisons post‐test = NS. (b) Fold change in the percentage of polyfunctional CD8 T cells (peptides / no peptides) of CD8 T cells treated as in (a). 2‐way ANOVA = NS. 0Gy MCMV+ vs. 4Gy MCMV+ groups in Tukey multiple comparisons post‐test = NS. (c‐d) Data shown from a single cohort. (c) Percent of NS4B tet + CD8 T cells in PBMC on day 8 post‐WNV infection. 1‐way ANOVA: *P*‐value = <0.0001. 0Gy MCMV+ vs. 4Gy MCMV+ groups in Tukey multiple comparisons post‐test = NS. (d‐f) Data shown from combined cohorts normalized to 0Gy (no WBI) MCMV(+) mice, all ANOVA post‐tests = NS. (d) NS4B tet + CD8 T cells in PBMC on day 8 post‐WNV. (e) Percent of NS4B tet + T cells that are granzyme B‐hi on day 8 post‐WNV infection. (f) Percent of NS4B tet + T cells that are Ki‐67‐hi on day 8 post‐WNV infection

To examine functional T‐cell responses, at 45 days post‐vaccination, PBMCs were harvested and stimulated with WNV peptides recognized by CD8 T cells in the presence of Brefeldin A. Only slight, non‐significant differences were noted across WBI doses and MCMV status with regard to individual cytokine production (Figure 4a, (Pugh et al., 2016)). Likewise, there were no significant trends in polyfunctional cytokine production in response to WNV‐specific peptides due to early‐life WBI dose (Figure 4b, (Pugh et al., 2016)). Finally, at the height of WNV challenge, 8‐days post‐WNV infection, NS4b + populations responded with roughly equivalent size (Figure 4c,d, (Pugh et al., 2016)), Granzyme B production (Figure 4e), and proliferation (Figure 4f) regardless of life‐long MCMV, WBI in youth, or both. Notably, adult vaccinated control groups responded with minimal NS4b + T cells (Figure 4c), likely highlighting their superior antibody control of the virus (Figure 3g).

### 2.4 WNV infection causes MCMV reactivation

**FIGURE 5:**
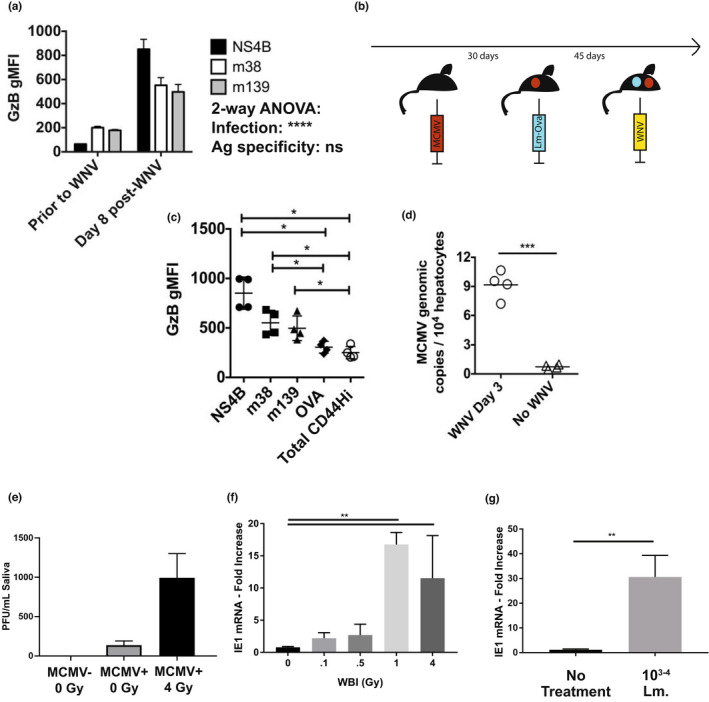
WNV infection causes reactivation of MCMV in adult mice. (a) Geometric MFI of granzyme B in tetramer‐specific CD8 T‐cell populations in PBMC from *n* = 4 adult MCMV(+) (latent) mice prior to WNV infection, and on day 8 post‐WNV infection. Tukey post‐tests between tetramers = ns. (b) Experimental design for data shown in (c). Mice were infected IP with 200 pfu smith strain MCMV, then rested for 30 days. Mice were then infected IV with 10^5^ cfu *listeria monocytogenes* expressing OVA epitope (LM‐ova). Memory populations were allowed to mature for 45 days. Mice were then infected with 2000 pfu WNV IP. (c) Geometric MFI of granzyme B in tetramer‐specific CD8 T cell populations from splenocytes collected on day 8 post‐WNV infection from mice in experiment (b). CD44hi = the remainder of tetramer non‐specific, CD44‐hi T cells. Shown are the results of Bonferroni post‐tests. (d) Genomic copies of MCMV from livers of mice with latent MCMV, either without WNV, or on day 3 post‐WNV infection, from qPCR for MCMV genomic DNA. (e‐g) 90 days after primary MCMV infection from 3 months of age, MCMV genomes were recovered from saliva day 3 post 4Gy irradiation and (f) transcription of MCMV *IE1* was measured in the salivary gland 3 days after a titrating dose of irradiation (as indicated) or after (g) infection with 10^3–4^ CFU of *listeria monocytogenes* (Mann–Whitney)

As expected, latent MCMV was reactivated by WBI in a dose‐dependent manner, across a variety of tissues (Sacher et al., 2008) (Figure S4A–D), with the highest reactivation seen at 4Gy. Prolonged immunological marks of reactivation were seen as a significant increase in representation of m139+ CD8 T cells on d 30 post‐WBI between unirradiated, 2Gy, and 4Gy group in an irradiation dose‐dependent manner. Because primary and memory immunity to vaccination were not altered by WBI, but survival from WNV was worsened specifically in the MCMV(+) 4Gy group (Figure 2), we wanted to examine the potential interaction of WNV and MCMV co‐infection. MCMV is kept latent by a combination of immune factors, including antibody, NK, and CD8 T cells (Doom & Hill, 2008). Low‐level MCMV reactivation may be traceable through populations of MCMV‐specific expanding CD8+ T cells (Karrer et al., 2003). m139 and m38 tetramer positive (m139+ and m38+) CD8 T cells are two such MCMV‐specific CD8+ populations that undergo life‐long expansion in latently infected mice (Munks et al., 2006). We found that both m139+ and m38+ CD8+ T cells increased GzB production during the height of WNV infection (Figure 5a).

We wanted to test the hypothesis that acute infection causes opportunistic reactivation of MCMV, as detected by increased activity in the MCMV tetramer‐specific memory T‐cell population. However, we first needed to create a control population of memory T cells to rule out bystander activation in the MCMV‐specific T‐cell response. To create both MCMV‐specific and control bystander populations of pMHC tetramer‐traceable T cells, we serially infected young mice (Figure 5b). Mice were first infected with MCMV. After MCMV latency, mice were infected with Listeria monocytogenes genetically modified to express the SIINFEKL epitope (LM‐Ova), to create a control bystander memory population. After the LM‐Ova response matured into memory, mice were infected with WNV, and cells analyzed for effector responses by pMHC‐Tet + GzB+ staining. In order to control for possible crossreactive or bystander response of MCMV‐specific memory T cell to a mimetic antigen found in WNV, two distinct MCMV‐specific memory populations were examined, defined by m38+ and m139+ tetramers, respectively.

As expected, GzB content was highest in NS4b + (WNV‐specific) T cells at the height of T‐cell response to WNV infection. However, GzB was significantly increased in in both m38+ and m139+ T cells, but not in bystander Ova memory T cells, nor in the entire remainder of CD44‐hi (memory pool) T cells (Figure 5c). Finally, to directly establish MCMV reactivation during WNV co‐infection, we collected liver in mice with latent MCMV on Day 3 following WNV infection. MCMV+ genomic content by qPCR was approximately 10‐fold higher in hepatocytes during WNV infection, compared with hepatocytes in mice that were mock‐infected with WNV (Figure 5d). To further validate these results, we examined other parameters of reactivation following varying doses of irradiation in the salivary gland. These included the salivary shed of virions (Figure 5e) measured as viral genomes (DNA), and the transcriptional activity within the tissue of the salivary gland (Figure 5f) measured via the viral *IE1* mRNA product. In these experiments, as well as during infection with the unrelated bacterial pathogen, *Listeria monocytogenes* (Figure 5g), increased transcription of the viral factor *IE1* and shed of virus in the saliva were detected. Therefore, latent MCMV was readily reactivated by various stressors, including both bacterial and WNV infection.

### 2.5 MCMV‐specific immunity is eroded in old mice that harbored latent MCMV during high‐dose, sub‐lethal WBI in youth

**FIGURE 6:**
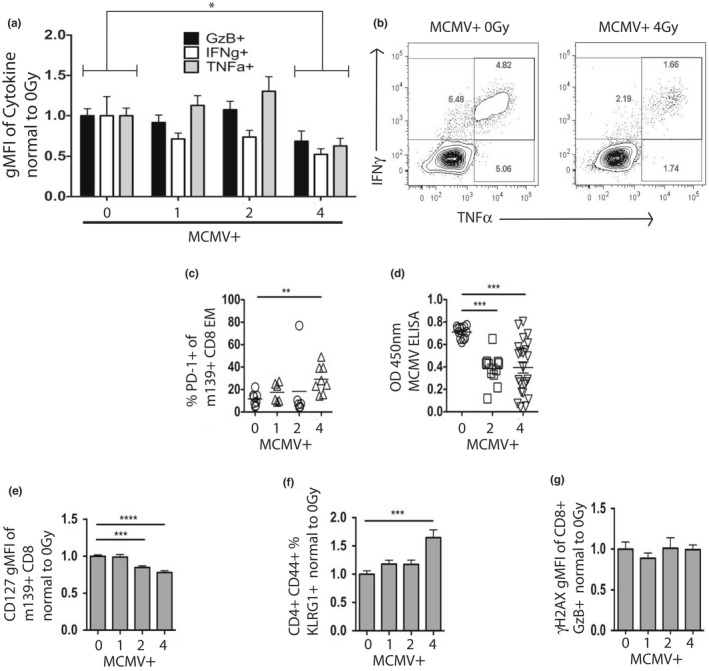
High‐dose sub‐lethal WBI in youth in MCMV+ mice taxes MCMV‐specific immunity. X‐axis: 0, 1, 2, and 4 = life‐long MCMV plus 0, 1, 2, and 4Gy in youth. (a‐b) Splenocytes from MCMV+ mice at 13 months of age were stimulated for 6 h with m139 peptide in the presence of brefeldin‐a. (a) Percent of individual cytokines normalized to 0Gy (no WBI) MCMV+ group. 2‐way ANOVA with Sidak's post‐test, 0Gy vs. 4Gy, *p* = 0.03. (B) Representative flow plots, as in (a). (c) Percent of PD‐1+ cells from PBMC at 13 months of age. Statistical outlier shown but not included in statistical analysis. (d) Results of MCMV ELISA from plasma collected at 19 months of age. (e) m139+ CD127 MFI of PBMC at 19 months of age from combined cohorts, normalized to 0Gy (no WBI) MCMV+ mice. (f) KLRG1‐hi percent of memory CD4 T cells from PBMC at 19 months of age from combined cohorts, normalized to 0Gy (no WBI) MCMV+ mice. (g) Gamma‐H2AX MFI of GzB+ CD8+ splenocytes at 13 months of age, normalized to 0Gy (no WBI) MCMV+ mice. (c‐g) Results of Dunnet's post‐tests shown

Given the propensity of WBI to cause MCMV‐reactivation (Sacher et al., 2008) (Figure S4 and Figure 5d,e), we wanted to examine MCMV‐specific immunity following WBI and repopulation. Our experimental design included a cross‐sectional harvest at 13 months of age (Figure 1), allowing us to measure MCMV‐specific immunity in middle‐life that could possibly inform on the outcomes of those mice remaining at 19 months. At 13 months of age, splenocytes were harvested and subjected to peptide stimulation with m139 peptide in the presence of Brefeldin A. CD8 T cells from mice who received 4Gy of WBI in youth produced lower amounts each of Granzyme B, IFNγ, and TNFα following peptide stimulation, compared with other, lower WBI doses (Figure 6a,b). MCMV(+) 4Gy mice further began to display increased intensity of PD‐1, an activation/exhaustion marker, on m139+ CD‐8 T cells by 13 months of age (Figure 6c). In those mice remaining at 19 months of age, WBI in youth resulted in a suppressed MCMV‐specific serum Ab production (Figure 6d). CD127 expression is known to decrease in exhausted populations of CD8 T cells in some chronic infections (Boettler et al., 2006; Tzeng et al., 2012). We found that WBI in youth decreased CD127 expression in a dose‐dependent manner in m139+ CD8 T cells by 19 months of age (Figure 6e). KLRG1 is a marker whose expression on memory CD4 T cells correlates with replicative senescence (Beyersdorf et al., 2007). The KLRG1‐high portion of memory CD4 T cells increased significantly in MCMV(+) 4Gy group by 19 months of age (Figure 6f). Neither marker of senescence/exhaustion was apparent in MCMV(−) mice regardless of WBI dose in youth (Figure S5A,B), strongly implying that these effects were mediated by MCMV reactivation, not WBI alone. To examine whether lasting DNA damage could be behind these effects, we analyzed levels of γH2AX in CD8+ T cells, a marker that labels double‐strand DNA breaks currently under repair. We found no increase in standing DNA damage in any group at 13 months (Figure 6g, Figure S5C), implying that the signs of exhausted/senescent anti‐MCMV response were not mediated by lasting WBI‐induced DNA damage. Moreover, at a steady state, 8 months post‐irradiation (13 months of life), there were no signs of increased MCMV DNA replication, suggesting stable latency regardless of prior irradiation in youth (Figure S5D).

### 2.6 WNV‐mediated CMV reactivation critically predicts lethal outcome

**FIGURE 7:**
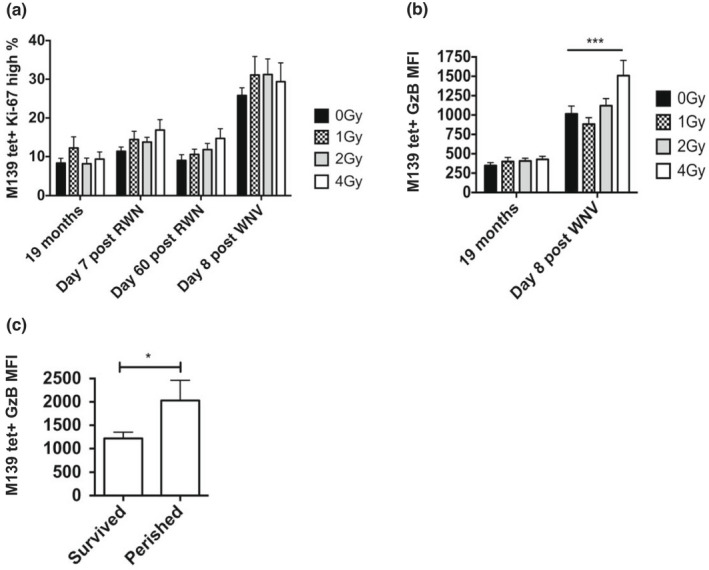
MCMV reactivation occurs in old mice during WNV infection, and is associated with increased mortality. Numbers on x‐axis indicate dose of WBI in youth in Gy. All mice MCMV(+), 19 months = MCMV(+) and non‐RWN‐vaccinated. (a) Percentage of Ki‐67 hi CD8 T cells within the m139 tetramer‐specific population in PBMC at various time points in late life. (b) Geometric granzyme B MFI within the m139 tetramer‐specific PBMC population at 19 months or on day 8 post‐WNV infection. ANOVA of 19 months = ns. Shown is the result of Bonferroni post‐test between radiation groups. (c) Geometric MFI of granzyme B in mice from the MCMV(+) 4Gy group in m139 tetramer‐specific PBMC population, stratified between mice that survived and mice that perished from WNV challenge

We examined proliferation and differentiation of m139‐specific CD8+ T‐cell populations during RWN vaccination and WNV challenge in mice that have received different doses of WBI in youth. At 19 months of age and prior to vaccination, numbers of m139‐specific CD8+ T cells in blood were not significantly different due to WBI dose in youth (Figure S5F). Regardless of radiation dose, m139‐specific populations exhibited similar (8–17%) fraction of dividing (Ki‐67+) cells at 19 months of life, 7‐days post‐RWN vaccination, and 60 days post‐RWN vaccination (Figure 7a). However, m139+ populations were significantly more prolific (28–35% Ki‐67+) in old mice on Day‐8 post‐WNV challenge regardless of prior irradiation (Figure 7a), indicating that MCMV reactivation occurred in old vaccinated mice during WNV infection, and that it was relatively independent of irradiation dose in youth. That was consistent with increased GzB production, which was enhanced twofold in the m139+ population during WNV challenge across WBI doses, and disproportionately so (threefold, *p* < 0.001) in the group MCMV(+) 4Gy, which exhibited decreased survival (Figure 7b). Upon detailed examination of the MCMV(+) 4Gy group, we could precisely stratify it by GzB levels in m139‐specific CD8 T cells into high‐ and low‐expressors; those mice that perished from WNV challenge had significantly higher levels (>1500 relative MFI; Figure 7c) of m139+ cells during the WNV response, compared with those that survived. By contrast, no parameters of anti‐WNV immunity correlated with death in the MCMV+ 4Gy group (see Figure 6). Rather, mice that perished from WNV appeared to have enhanced MCMV‐specific T‐cell responses (Figure 7c), though WNV‐specific antibody was equivalently neutralizing (Figure S5E).

Overall, our data support a model in which MCMV reactivation during WNV co‐infection increases the likelihood of lethal outcome when coupled with sub‐lethal WBI in youth. Two lines of evidence support this conclusion: (i) measurable defects in MCMV‐specific immunity were observed in the MCMV(+) 4Gy group (Figure 6), allowing for enhanced MCMV reactivation (Figure 7c) and possibly inflammation during WNV infection, ultimately leading to increased mortality from co‐infection by mechanisms that are under investigation; (ii) by contrast, WNV‐specific immunity was functionally equivalent regardless of CMV status or radiation dose (Figures 3, 4, 5). These results are discussed below.

## 3 DISCUSSION

In our prior work (Pugh et al., 2016), we found no evidence that a single exposure to ionizing radiation of up to 4Gy in youth causes immune defects in old age. However, as irradiation is known to reactivate persistent pathogens (including CMV), and as most of the population carries this virus, we elected to repeat these experiments in tandem with a primary persistent cytomegalovirus infection (which is ubiquitous in human populations) and examined the contribution of this pathogen to immune modulation following an ionizing radiation event in youth. We found that following WNV vaccination and challenge, old mice with MCMV that received 4Gy irradiation in youth exhibited worse survival than any other group of animals, whereas neither high‐dose WBI or MCMV alone made either the survival or the immune responses any worse compared with aging alone.

To elucidate the basis behind this surprising relationship, we carefully examined WNV immunity and found no differences between groups with high‐ and low‐mortality in humoral or cellular immune responses. Because increased mortality was exclusive to the MCMV(+) 4Gy group, we reasoned that an MCMV‐specific immune defect, linked to high WBI, would be a likely culprit. It was possible that 4Gy WBI generally weakened memory cells. However, memory T cells in MCMV(−) 4Gy mice did not display any markers of exhaustion or senescence following repopulation. Therefore, it was unlikely that repopulation stress alone caused MCMV‐specific immune defects. We next suspected that WBI may have triggered MCMV reactivation, and found that MCMV reactivated in a WBI‐dose dependent manner, such that the reactivation of MCMV was of greater magnitude and involves a broader array of tissues in 4Gy‐exposed mice compared to 2Gy‐exposed mice. Steady‐state levels of latent MCMV were no higher in 4Gy mice than in 0Gy mice following repopulation, and therefore, the MCMV burden was not permanently increased by WBI and reactivation. We further found that irradiated MCMV+ mice exhibited dose‐dependent alterations in MCMV‐specific T‐cell responses, which were most pronounced in 4Gy‐irradiated mice. Together with the finding that WNV infection also caused MCMV reactivation, these results suggested that the most likely scenario is that WBI weakened MCMV immunity through the systemic MCMV reactivation event following WBI, and that lifelong subclinical reactivations further potentiated this effect. Upon WNV infection, another MCMV reactivation challenged the MCMV response already weakened by prior irradiation, making animals susceptible to succumbing to a combination of WNV and MCMV. Further work is necessary to elucidate whether systemic reactivation without WBI is sufficient to weaken MCMV immunity, and whether memory T cells are more susceptible to activation‐induced exhaustion following WBI and repopulation.

The danger of co‐infection with MCMV and WNV is at first counter‐intuitive, in that Th1‐mediated responses to either virus should aid in clearing the other. Following this logic, latent gamma‐herpesvirus has been shown to be protective in the context of bacterial co‐infection in adult mice (Barton et al., 2007), and we have also shown that latent MCMV is protective in Listeria infection in old mice by broadening TCR repertoires (Smithey, PNAS). The increased mortality due to MCMV co‐infection is therefore specific to old mice. MCMV utilizes a variety of immune evasion mechanisms; however, we did not see evidence of reduced immune responsiveness in mice that died in our challenge experiments—in fact, we observed increased signs of immune reactivity against CMV and WNV, which is not consistent with immune evasion. If mortality from WNV is immunopathogenic in old mice, then increased immune activation from MCMV might increase mortality. However, WNV immune infiltrates in the brains of old immunocompetent mice are few compared with other immunopathogenic infections (Brien et al., 2009) making this scenario unlikely. It is possible that CMV could increasingly infect the CNS as a result of increased inflammation and WNV entry into the brain during co‐infection. The precise mechanism of MCMV and WNV co‐infectious mortality remains to be elucidated and experiments are in progress to address it conclusively.

Latent CMV can be found in a variety of tissues in both infected mice and humans (Boeckh & Geballe, 2011; Sacher et al., 2008). CMV reactivation can occur in association with cell differentiation, immune compromise, or tissue damage, and has recently been reported during co‐infection in a model of herpesvirus and helminth infection (Reese et al., 2014). The pathology of WNV includes viremia and infection of a variety of organs (Samuel & Diamond, 2006). It is therefore not surprising that WNV could reactivate CMV during the course of infection. We have confirmed that similar MCMV reactivation occurs in mouse models of Listeria infection. It remains to be seen what infectious burden if any is sufficient to reactivate CMV in humans. Our work points to the possibility of additional risks borne by older CMV+ individuals during systemic infections due to acute CMV reactivation. This also implies that the enhanced mortality associated with CMV is perhaps due to the cumulative effect of opportunistic CMV reactivation events during new infections throughout life, rather than the static immune burden of latent CMV. Individual history of systemic infection may therefore be an important predictor of mortality in CMV+ individuals.

Our results also imply that the trajectory of immune health in individuals exposed to WBI is highly dependent on CMV status at the time of exposure. Because CMV is highly prevalent in human populations, radiobiology studies should discriminate based on latent pathogen status, and radiobiology studies with animal models should include latent infections that closely mirror human populations. In these foundational cohorts, our experimental design utilized male mice to minimize the confounding influence of endocrine changes that occur during female sexual senescence at late‐life time points. Importantly, future cohorts will be comprised of female mice, and incorporate additional endocrine measures that may inform on the interplay of DNA damage, chronic infection, and immunity. Moreover, future work will be conducted to probe the contribution of WBI and CMV to the potential lethality of other clinically relevant microbes, such as SARS‐CoV2 and influenza.

## 4 METHODS

### 4.1 Mice

Adult (<6 month) male C57BL/6 mice were acquired from Jackson Laboratories and housed under specific pathogen‐free conditions in the animal facility at the University of Arizona (UA). All experiments were conducted by guidelines set by the UA Institutional Animal Care and Use Committee. As needed, mice were euthanized by isofluorane and spleen was collected into complete RPMI supplemented with 5 or 10% fetal bovine serum (FBS). Blood was taken from the heart or retro‐orbitally for cross‐sectional and longitudinal harvests, respectively, and red blood cells were hypotonically lysed.

### 4.2 Viruses and vaccine

Smith Strain MCMV was obtained as multiple passage stock from Drs Ann Hill (Oregon Health & Science University, Portland, OR) or as a low‐passage infectious clone from and Wayne Yokoyama (Washington University, St. Louis, MO). Animals were injected IP at 10^5^ pfu / mouse, and both stocks showed identical acute responses. West Nile Virus (WNV): Strain 385–99, a kind gift from Robert Tesh, was injected IP at 2000 pfu / mouse. Replivax WNV (kind gift from Drs P. Mason, N. Bourne and G. Milligan, U. Texas Med. Branch, Galveston, TX) was injected IP at 10^5^ pfu / mouse. Verification of viral titer and production of RWN stock are described elsewhere (Uhrlaub et al., 2011).

### 4.3 Peptide stimulation

Blood samples were taken at 45 days following RWN vaccination, hypotonically lysed, and stimulated ex vivo with a pool of NS4b 2488–2496 and E 347–354 peptides (21st century Biochemicals, Marlborough MA) both at 10^−6^ M. Stimulation took place over 6 h in the presence of BFA. Splenocytes from 13‐month‐old mice during cross‐sectional harvest were treated identically for 5 h with 2 ng/μl MCMV m139 peptide.

### 4.4 MCMV ELISA

We used commercial MCMV ELISA kits (Cat# IM‐811C, XpressBio, Thermont, MD). Serum samples were applied at 1:50, 1:250, and 1:500 dilutions in duplicate, and at 1:50 in wells coated with uninfected cell lysate (negative control). MCMV‐mouse serum was used as negative biological control. Serum AB was detected with anti‐mouse HRP‐bound AB in an enzymatic reaction, and OD was read at 450 nm. Control wells for each mouse were subtracted from OD.

### 4.5 Plaque reduction neutralization test (PRNT)

Serial dilutions of mouse serum (1:10 minimum) were incubated with 100 pfu / well live WNV from the same stock received by mice, in a 96 well format, for 6 h at 4°C. Samples were then applied to a monolayer of Vero cells also in 96 well format, and allowed to incubate at 37°C with 5% CO_2_ for 25 h. Resulting monolayers were fixed with ice cold 50% acetone 50% methanol for 30 min at −20°C, and allowed to dry overnight. Resulting monolayers were assayed with anti‐WNV antibody clone EG16, a kind gift from Michael Diamond, followed by peroxidase labeled Goat Anti‐mouse IgG (XPL, inc, Gaithersburg MD). Infectious lesions were visualized in a DAB reaction. The dilution factor necessary for 90% reduction of infectious lesions was established by hand count. The average of duplicate assays per mouse was used.

### 4.6 Irradiation

Whole‐body irradiation was performed on a Gammacell Cs^137^ source irradiator calibrated by in‐house physicist from the UA Health Sciences Center. Dosage was verified with thermal luminescence dosimeters (TLD) (Landauer Inc., Glenwood, IL) and TLDs from the Medical Radiation Research Center at the University of Wisconsin. Dosages fell within 5% of expected values. Effective dose rate ranged 70.2–68.36 cGy/min depending on the age of the source, and distance. For whole body irradiation, a maximum of 8 mice were placed in sterile RadDisks (Braintree Scientific, Braintree, MA) with no separation. WBI occurred before noon on a light–dark cycle 7 AM‐7 PM.

### 4.7 Flow cytometry

Prior to each collection, voltages were manually calibrated to a common template using Rainbow Beads (BD Biosciences, San Jose, CA), to insure accurate MFI tracking over time. Fluorescent conjugated α‐Mouse antibodies against CD3(SK7), CD4(MCD0430), CD8a(S3‐6.7), CD62L(MEL‐14), CD44(IM7), α‐Ki‐67(B56), CD127(A7R34), KLRG1(2FI), CD86(GL‐1), B220(RM2630), NK1.1(PK136), CD49b(DX5), CD19(RM7717), IgM(II/41), MHC‐ii(M5/114.15.2), were purchased from commercial sources. Tetramers against NS4b (H‐2D[b] – SSVWNATTA), m139 (H2‐K[b] ‐ TVYGFCLL), m38 (H‐2 K[b] – SSPPMFRV), and Ova (H‐2 K[b] – SIINFEKL) were obtained from the National Institutes of Health Tetramer Core Facility. Staining occurred at 4C followed by fixation and permeabilization (FoxP3 kit, eBioscience). Blood and spleen counts occurred on a Hemavet cell counter (Drew Scientific, Dallas, TX). Samples were run on a Fortessa Flow Cytometer equipped with 4 lasers and using DiVa software (BD Biosciences). Compensation and analysis were performed using FlowJo software (Tree Star, Ashland, OR).

### 4.8 qPCR

Tissues were harvested and placed into Eppendorf tubes with 1 ml Tri‐Reagent (Life Technologies/Ambion, Grand Island, NY) and sterile 1 mm silica beads (Biospec Products, Bartlesville, OK), and immediately frozen in a dry‐ice and 100% ethanol bath. Samples were thawed, bead‐beaten, and DNA was extracted with phenol‐chloroform. In the case of liver samples, DNA was subjected to two consecutive rounds of phenol‐chloroform extraction. Samples were normalized for DNA content and subjected to qPCR in quadruplicate with SYBR Green master mix (Life Technologies). Primers for either MCMV IE1 (*IE1‐1*: CCC TCT CCT AAC TCT CCC TTT, *IE1‐2*: TGG TGC TCT TTT CCC GTG) or C57BL/6 beta‐actin (*BA‐1*: AGC TCA TTG TAG AAG GTG TGG, *BA‐2*: GGT GGG AAT GGG TCA GAA G) were used. Serial dilutions of plasmids (pCR‐Blunt) with either IE1 or beta‐actin insertion sequences were used in each plate to establish real counts and primer efficiencies. Primers and plasmids were developed by Bijal Parikh, and plasmids were supplied as a kind gift from Wayne Yokoyama. Samples were run on ABI 7900 (Life Technologies/Applied Biosystems) at the University of Arizona Genomics Core facility. Alternatively, tissues were harvested from mCMV infected and uninfected age‐matched controls and collected into microcentrifuge tubes containing 5x volumes RNAlater (Millipore Sigma) and stored at −80. Each sample was thawed and processed using Nucleospin RNA plus with DNA removal (Machery‐Nagel) preps per manufacturer's protocol. Reverse transcription was performed using OmniScript reverse transcriptase (Qiagen) and oligo‐dT primers. qPCR to measure the expression *β‐actin* and *IE1* of was performed using PowerUP SYBR Green Master Mix (Applied Biosciences) on a Step One real‐time PCR system (Applied Biosciences) using the following cycle protocol: an initial step at 2 min 50°C followed by 95° for 10 min, followed by 40 cycles of 95° for 15 s, 60° for 1 min. Sample RNA content was normalized to *β‐actin* and expression of *IE1* was compared using the 2^−ΔΔCT^ method. Cycle 32 was set as a negative cut‐off based on uninfected controls. Primer sets were gifted by Chris Benedict, PhD, La Jolla Institute of Immunology.

### 4.9 Statistics

Statistical analysis was performed using Prism 6.0 (GraphPad Software). When data from multiple cohorts were combined for analysis, data from each cohort were normalized to the average of M0 group from that same cohort, to avoid cohort‐specific biases. Significance is noted as follows throughout: ns = not significant, ****p < 0.0001, ***p < 0.001, **p < 0.01, *p < 0.05. All error bars shown are SEM.

### 4.10 Data

Data comprising all of the main figures are available in Appendix S2.

## AUTHOR CONTRIBUTIONS

JLP designed and performed experiments, and wrote the manuscript. CPC performed experiments. ASS performed experiments. JLU performed experiments and designed assays. JP‐T performed experiments. T.H. and K.N. provided critical advice. JN‐Ž designed experiments, directed the study, wrote, and edited manuscript.

## CONFLICT OF INTEREST

The authors have no conflicts of interest to declare.

## Supporting information


Appendix S1

Appendix S2

## Data Availability

Data comprising all main figures is included in Supplementary Data file 6.
