# Supplementary material for: Lifelong cytomegalovirus and early‐LIFE irradiation synergistically potentiate age‐related defects in response to vaccination and infection
Source: Aging Cell. 2022 Jun 3;21(7):e13648. doi: 10.1111/acel.13648 (PMC9282846; doi:10.1111/acel.13648)
Supplement: Supplementary file 1 — Appendix S1 [file ACEL-21-e13648-s002.pdf]

FIGURE S1

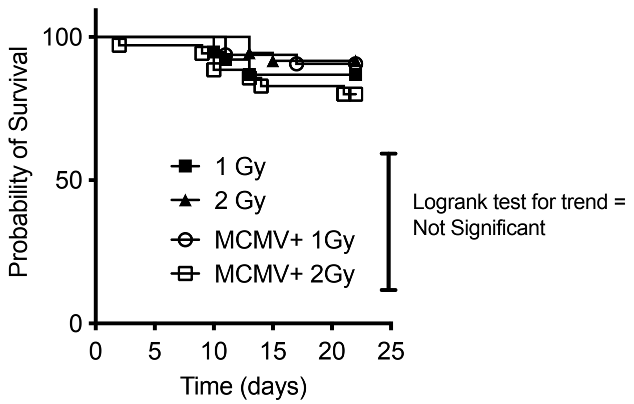

Figure S1. 1 or 2 Gy WBI in youth, alone or in combi-nation with MCMV infection, does not significantly effect survival from WNV challenge in old age. Survival shown following RWN vaccination at approximately 19 months of age, and WNV challenge at approximately 21 months of age. Mice were infected with 2000 pfu WNV IP. Graph is a combination of both cohorts (no group statis-tically different between cohorts). Groups of mice infected or not with MCMV, and or irradiated with 1 or 2 Gy WBI are not signifi-cantly different, nor is there a significant

Figure S2

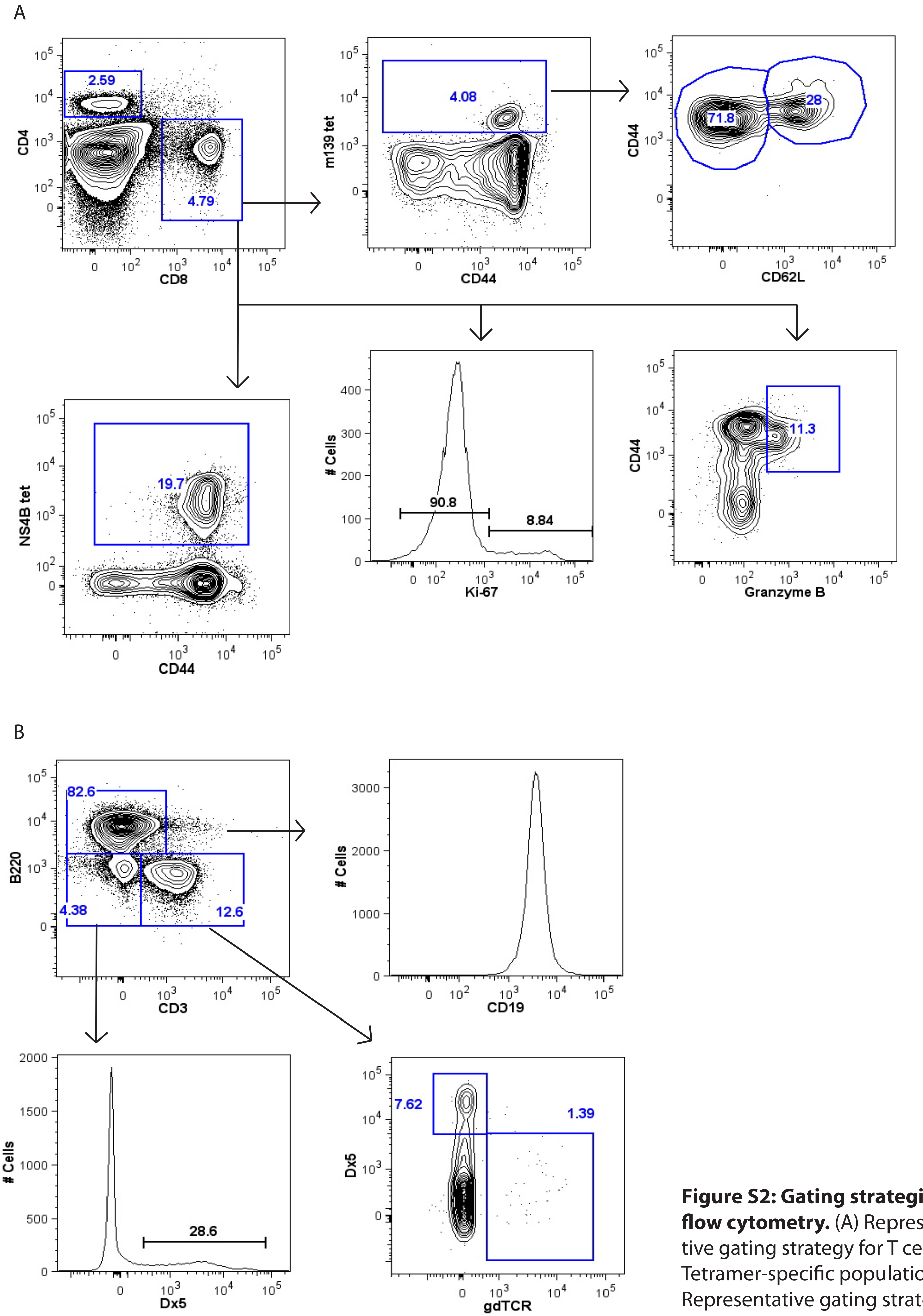

Figure S2: Gating strategies for flow cytometry. (A) Representative gating strategy for T cells and Tetramer-specific populations. (B) Representative gating strategy for B, NK, γδT and NKT cells.

Figure S3

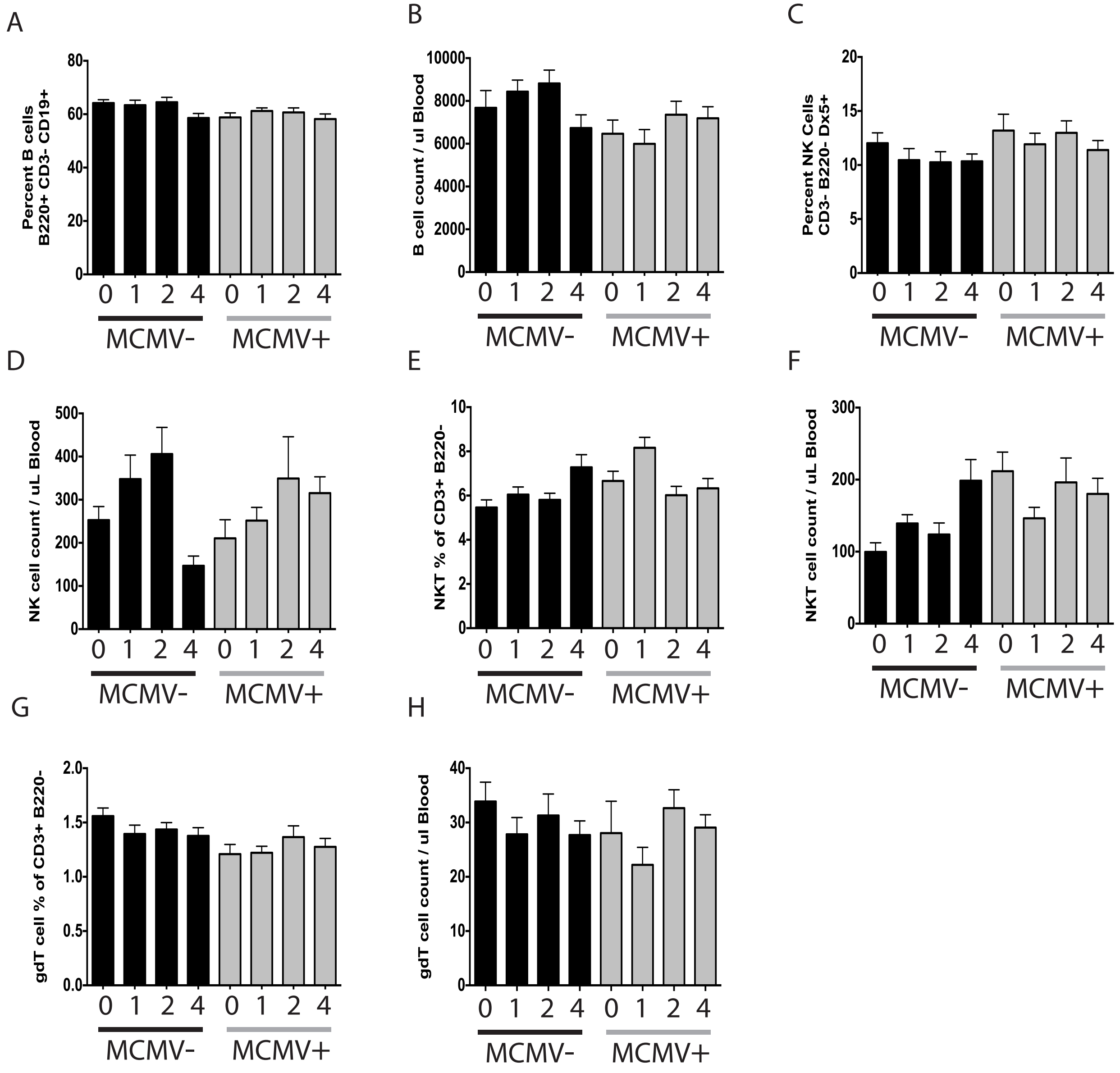

Figure S3: Standing counts and proportions of cells in PBMC of combined cohorts at 19 months of age. M indicates mice that have been infected with MCMV for life. Numbers indicate amout of WBI received in youth in Gy. (A) Proportion of B cells. (B) Count of B cells. (C) Proportion of NK cells. (D) Count of NK cells. (E) Proportion of NKT cells. (F) Count of NKT cells. (G) Proportion of γδT cells. (H) Count of γδT cells.

Figure S4

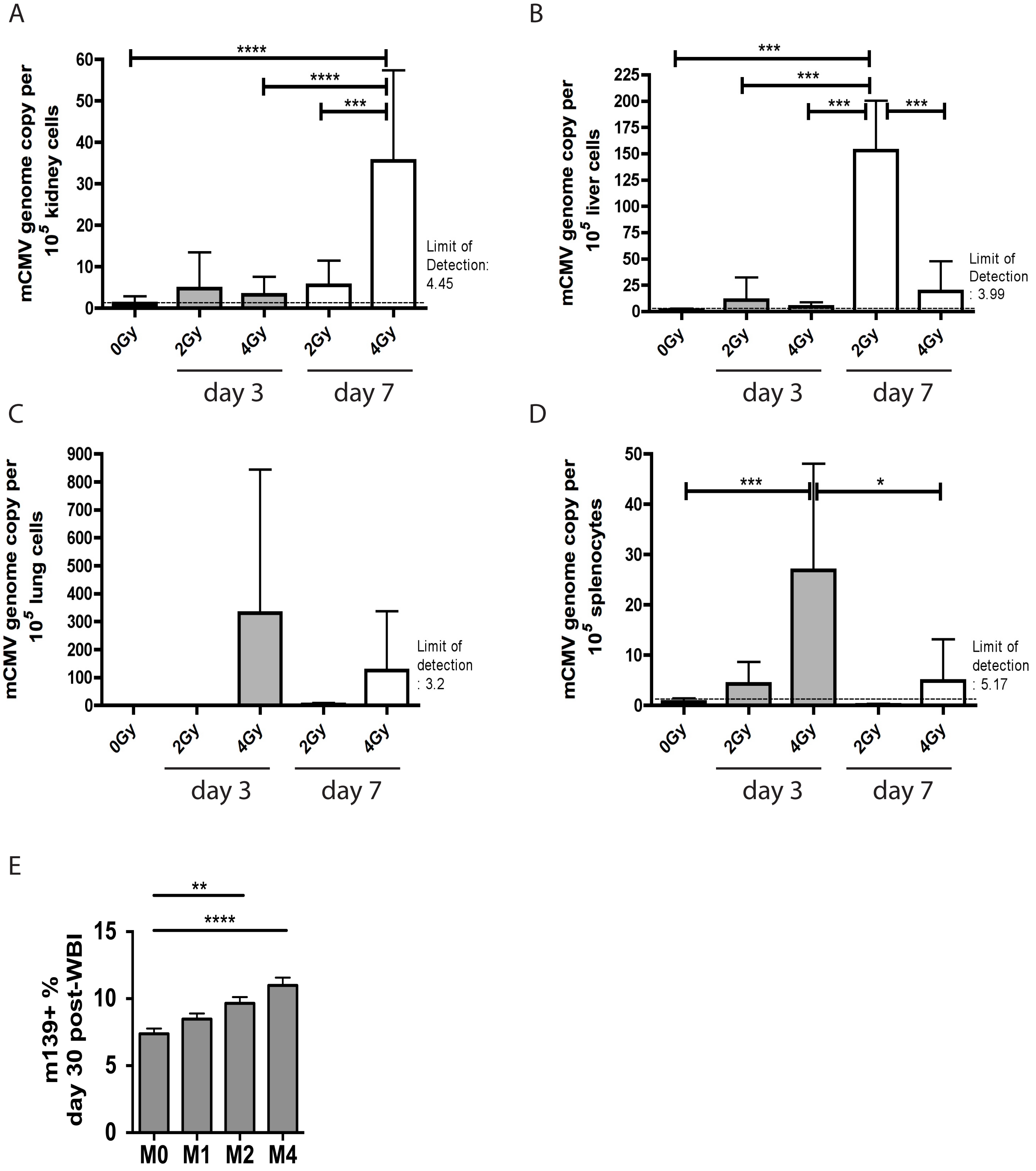

Figure S4: WBI reactivates MCMV. MCMV DNA assayed by qPCR from various tissues, normalized to signal from mouse Actin gene. All mice were adult C57BL/6 male mice subjected to WBI (2Gy or 4Gy) or no WBI (0Gy) 60+ days post-MCMV infection (latent MCMV). Tissues were harvested either 3 days or 7 days post-WBI. (A) Increase in MCMV genome copies in the kidney. (B) Increase in MCMV genome copies in the liver. (C) Increase in MCMV genome copies in the lung. (D) Increase in MCMV genome copies in the spleen. Shown are the results of Bonferroni post-tests. (E) m139+ proportion of CD8 T cells from PBMC of MCMV+ mice 30 days post-WBI. Shown are the results of Dunnet's post-test.

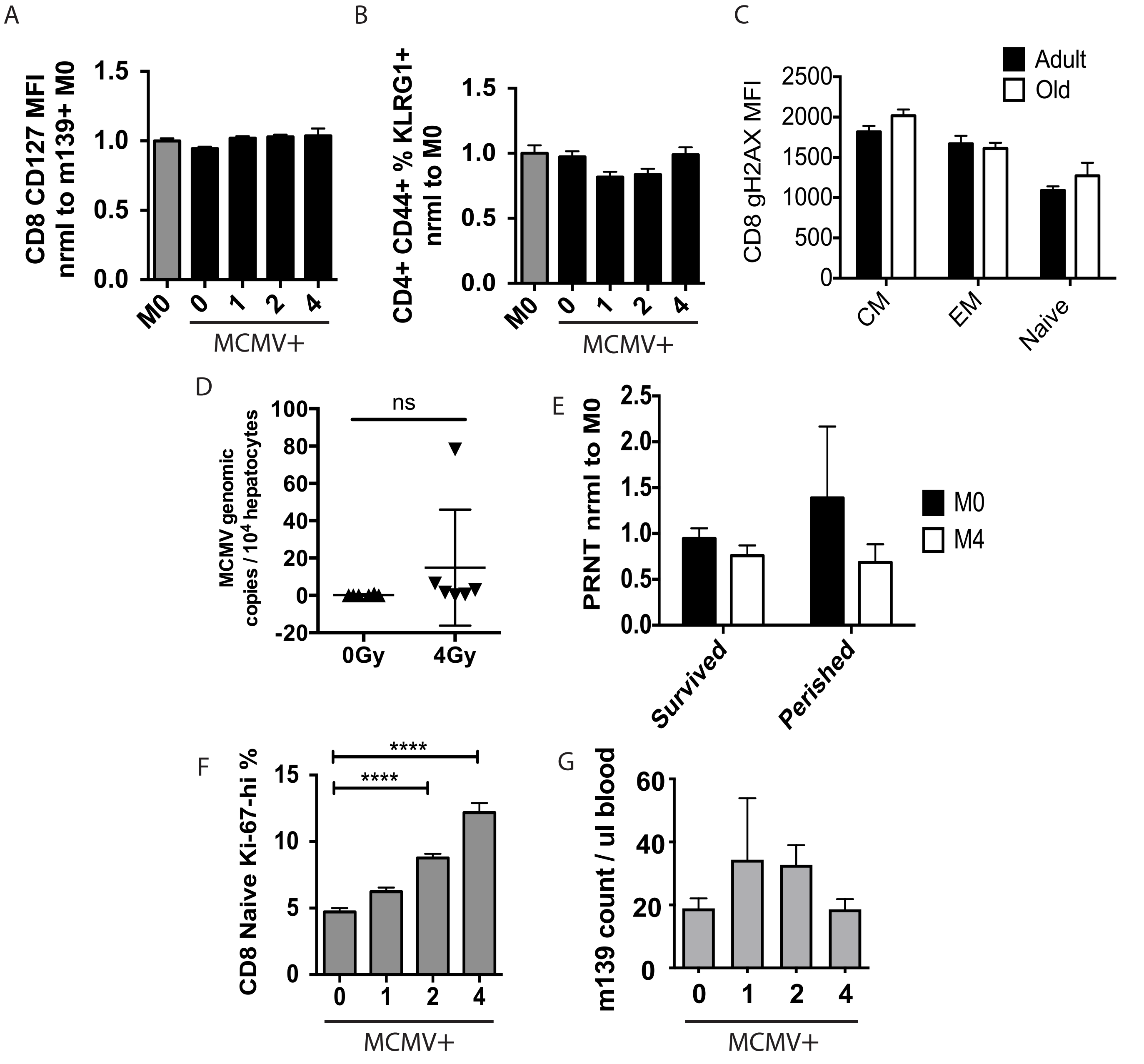

Figure S5: WBI alone does not increase senescence markers or latent MCMV burden, aging does not increase standing DNA damage in immune cells, and PRNT doesn't correlate with death from WNV. M0 = MCMV(+) 0Gy, M4 = MCMV(+) 4Gy. (A) CD127 MFI in CD8 T cells of mice at 19 months of age from PBMC of combined cohorts, normalized to M0 group. (B) KLRG1+ proportion of memory (CD44-hi) CD4 T cells at 19 months of age from PBMC of combined cohorts, normalized to M0 group. (C) γH2AX MFI of adult (~5 months) and old (>18 months) male C57BL/6 mice in CD8 T cells from spleen. n=8 per age group. (D) MCMV genomic copies in hepatocytes at 13 months of age from mice with life-long MCMV subjected to WBI at 5 months age. Results of Mann-Whitney test shown (E) PRNT 90% titer reduction normalized to M0 survivors. Statistical post-tests are non-significant. (F) CD8 Naive Ki-67 hi portions in MCMV(+) mice from PBMCs 30 days post-WBI. (G) m139-tetramer specific CD8+ T cell counts from blood at 19 months of age in MCMV(+) mice. ANOVA = ns. Tukey's multiple comparison tests between all groups = ns.
